# Comparison of Alpha Blockers in Treatment of Premature Ejaculation: A Pilot Clinical Trial

**DOI:** 10.5812/ircmj.13805

**Published:** 2013-10-05

**Authors:** Yigit Akin, Hakan Gulmez, Mutlu Ates, Aliseydi Bozkurt, Baris Nuhoglu

**Affiliations:** 1 Harran University School of Medicine, Department of Urology, Sanliurfa, Turkey; 2 Department of Family Medicine, Baskent University School of Medicine, Ankara, Turkey; 3 Department of Urology, Afyon Kocatepe University School of Medicine, Afyonkarahisar, Turkey

**Keywords:** Premature Ejaculation, Quality of Life, Serotonin Uptake Inhibitors

## Abstract

**Background:**

Premature ejaculation (PE) is the most common sexual disorder in men and studies reported prevalence up to 30% (1, 2). PE is not a life-threatening medical condition but it influences the quality of life (QoL).

**Objectives:**

The aim of this study was to compare the efficiency, and safety of alpha blocker drugs in the treatment of patients with premature ejaculation (PE). Additionally we investigated the quality of life (QoL) in patients with PE who were treated with alpha blocker drugs.

**Materials and Methods:**

This study was a pilot clinical trial. Prospectively documented 108 patients with PE were treated and were followed-up in urology outpatient clinic. All patients were divided into 5 groups according to used alpha blocker agents which were determined by simple randomization. Silodosin 4mg (Group 1, n = 21), tamsulosin hydrochloride 0.4mg (Group 2, n = 23), alfuzosin 10mg (Group 3, n = 22), terazosin 5mg (Group 4, n = 21), doksazosin mesylate 4mg (Group5, n = 21), were used for treatment. The demographic parameters of patients, pre and post treatment intravaginal ejaculation latency time (IELT), PE Profile (PEP), and QoL index were recorded and evaluated. Effectiveness of treatment was evaluated by measuring IELT. Additionally, side effects of drugs were recorded. P < 0.05 was considered statistically significant.

**Results:**

All alpha blocker drugs were statistically effective for preventing PE. Notably, silodosin seemed to be more effective for preventing PE than other alpha blockers (P < 0.05). However all alpha blockers provided development in QoL scores, silodosin was a little better than other drugs in statistical analyses. Furthermore statistical increase in IELT and decrease in PEP were provided more in Group 1 than other groups (P < 0.05).

**Conclusions:**

Silodosin seems to be able to even more prevent PE. Silodosin may provide development in QoL than other alpha blocker agents. Additionally, lower systemic adverse events and more effectivity are the prominent features of silodosin in PE.This study was a pilot clinical trial. Prospectively documented 108 patients with PE were treated and were followed-up in urology outpatient clinic. All patients were divided into 5 groups according to used alpha blocker agents which were determined by simple randomization. Silodosin 4mg (Group 1, n = 21), tamsulosin hydrochloride 0.4mg (Group 2, n = 23), alfuzosin 10mg (Group 3, n = 22), terazosin 5mg (Group 4, n = 21), doksazosin mesylate 4mg (Group5, n = 21), were used for treatment. The demographic parameters of patients, pre and post treatment intravaginal ejaculation latency time (IELT), PE Profile (PEP), and QoL index were recorded and evaluated. Effectiveness of treatment was evaluated by measuring IELT. Additionally, side effects of drugs were recorded. P < 0.05 was considered statistically significant.

## 1. Background

Premature ejaculation (PE) is the most common sexual disorder in men and studies reported prevalence up to 30% (1, 2). PE is not a life-threatening medical condition but it influences the quality of life (QoL) (3). However there has not been an established universally accepted definition of PE, but according to the Diagnostic and Statistical Manual of Mental Disorders, 4th edition, text revision (DMS-IV-TR): “persistent or recurrent ejaculation with minimal stimulation before, on or shortly after penetration and before the person wishes it”, which is associated with “marked distress and interpersonal difficulty” is usually used worldwide for the description (4).

Up to the present, PE’s treatment options such as psychotherapy, behavioral and pharmacotherapy were described in literature (5). Although behavioral and psychological counseling are the first treatment options for PE, they require active participation of partners. Some cultural and socioeconomic groups do not participate in therapy (6). Patients with PE may want to solve this medical problem rapidly. Therefore pharmacotherapy comes into question.

However selective serotonin reuptake inhibitors (SSRIs) have not been directly designed for PE, side effects of SSRIs help to treat PE (7). Thus, SSRIs have been used for PE worldwide. SSRIs were designed for continuous usage. They are used for PE at least for 6 weeks, except dapoxetine (8). This regimen may encourage some adverse effects such as reduced libido, seratonergic syndrome such as mild headache, nausea, sweating, and dizziness (5). Therefore researches have been continued for more safe, effective and less adverse effective drugs in PE treatment. Seminal vesicles are important parts of ejaculation and alpha-1 adrenergic receptors present in seminal vesicles (9). Thus blocking the alpha receptors in seminal vesicles may provide a delayed ejaculation. However there are limited studies suggesting that alpha blockers could be used in the treatment of PE.

## 2. Objectives

According to our knowledge, a comparison between effectivity and safety of alpha blockers with QoL assessment for PE treatment still has not been reported in the literature. In this present study, we aimed to compare the efficiency, safety and influence ratio of QoL of alpha blocker agents in patients with PE. According to our knowledge our study is unique because more than two alpha blockers were compared in the treatment of PE in literature.

## 3. Materials and Methods

This study was a pilot clinical trial and open-labeled. All patients fully understood the treatment and aim of the study, and also written informed consent was obtained. Ethical approval for this study was obtained from our institute and ethics committee on 18th of September in 2012 (numbered: 2012/4145-874). Moreover patients’ privacy was ensured by ethical approvals. In our series, from October 2012 to April 2013, 111 heterosexual men with PE were evaluated. All of the participants were refractory to psychotherapy for PE or did not want any psychotherapy. Regularly followed 108 patients with PE were enrolled. All patients had met the following PE criteria, according to the DSM-IV-TR (4). Three patients were excluded for nonregular follow-up. Our study was performed in the city center of Konya, in Turkey. One million people live in the city center of Konya. According to 2012 data of census in Turkey, 340.000 males older than 18 years old, live in the center of Konya. According to literature, about 110.000 (about 30%) males had PE (1-2). We aimed to reach 1/1000 (110) of them for statistical analyses. Our data contained 108 patients. We used convenience sampling.

All patients were divided into 5 groups according to used alpha blocker agents. Agents were given by simple randomization. Group 1 (n = 21) included patients who used silodosin 4mg; Group 2 (n=23) included patients who used tamsulosin hydrochloride 0.4mg; Group 3 (n=22) included patients who used alfuzosin; Group 4 (n = 21) included patients who used terazosin 5 mg; Group 5 (n = 21) included patients who used doksazosin mesylate 4 mg. Demographic data included detailed physical examination and sexual history including history of PE, age (year), body mass index (kg/m2), comorbidities, heterosexuality, drug of abuse. Pre and post treatment intravaginal ejaculation latency time (IELT), PE Profile (PEP), and QoL index were recorded. Additionally side effects of alpha blockers were noted. In laboratory, routine biochemical tests, prostate specific antigen (if the patient was older than 45 years old), urine analysis were performed. Effectiveness of treatment was evaluated by measuring IELT. Exclusion criteria included drug of abuse, homosexuality, sexual disorders, chronic prostatitis, noncompliance of DSM-IV-TR, nonregular follow-up, still using drugs such as serotonin reuptake inhibitors for PE (4). All of the alpha blocker agents were used at least 14 days continuously. IELT, PEP, and QoL scores were calculated before treatment as baseline. The first IELT was calculated after the 3^rd^ day of treatment. The aim of these calculations was to measure IELT after providing pharmacological efficiency of alpha blocker drugs in serum (10). The second IELT was calculated at the 7^th^ day of treatment, and the third IELT was provided at the 14^th^ day of treatment. The average of these IELTs were calculated and used as mean IELT. At the end of the 14th day of treatment PEP and QoL forms were filled by all participants. Descriptive results were reported for all studied parameters. Independent samples T test and paired samples T test were used for statistical analysis. We used sample randomization for drug use. Additionally, we used one way ANOVA test for checking the normal assumption. Statistical significance was considered P < 0.05 and all p values were 2-sided. All statistical analyses were performed with the Statistical Package for Social Sciences (SPSS) for Windows 16.0 (SPSS Inc., Chicago, IL). Graphics were plotted using Microsoft Excel.

## 4. Results

Participants had a mean age of 45.8 ± 9.7 years (range 20-75 years), and reported to have PE for an average of 2.43 ± 0.98 years (range 1-5 years). 

Pretreatment demographic data of groups were not statistically significant different in each other (Table 1). Additionally pretreatment IELT, PEP, and QoL scores were similar among groups (Table 1). 

**Table 1.** Pairwise Comparison of P Values in Groups’ Demographic Data

Parameters						P Values for Pairwise Comparisons									
						Group 1 vs. Group.				Group 2 vs. Group			Group 3 vs. Group		Group 4 vs. Group
	**Group 1 ** **Mean± SD**	**Group 2 ** **Mean± SD**	**Group 3 ** **Mean± SD**	**Group 4 ** **Mean± SD**	**Group 5 ** **Mean± SD**	**2**	**3**	**4**	**5**	**3**	**4**	**5**	**4**	**5**	**5**
**Age (Years)**	49.4 ± 11.8	43.3 ± 8.9	46 ± 8.6	44.5 ± 9.1	45.7 ± 9.4	0.06	0.29	0.14	0.27	0.29	0.64	0.38	0.58	0.91	0.68
**BMI (kg/m2)**	24.1 ± 2.5	24.8±2.5	25 ± 2.8	25.1±3.4	25.6 ± 3.6	0.4	0.28	0.3	0.13	0.77	0.73	0.38	0.93	0.54	0.64
**History of PE **	2.6 ± 1.01	2.1±0.9	2.5 ± 0.9	2.3 ± 0.9	2.5 ± .1.1	0.13	0.81	0.34	0.94	0.19	0.57	0.18	0.46	0.88	0.41

There was a statistical significant development in QoL, increase in IELT, and decrease in PEP scores in post treatment period for all groups (Table 2). 

**Table 2.** Comparisons of Groups According to the Baseline and Post Treatment Intravaginal Ejaculation Latency Time, Premature Ejaculation Profile, and Quality of Life[Table-fn fn5257]

Parameter	Mean ± SD Base IELT (second)	Mean ± SD Post Treatment IELT (second)	P Value	Mean ± SD Base PEP	Mean ± SD Post Treatment PEP	P Value	Mean ± SD Base QoL	Mean ± SD Post Treatment QoL	P Value
**Group 1**	18.8 ± 12.93	151 ± 53.9	< 0.001^b^	12.4 ± 3.3	6.8 ± 2.1	< 0.001^b^	3.7 ± 0.7	2.1 ± 0.8	< 0.001^b^
**Group 2**	23 ± 12.8	89.7 ± 31.4	< 0.001^b^	13.7 ± 2.6	11.8 ± 2.3	< 0.001^b^	3.4 ± 0.8	2.3 ± 1.2	0.001^b^
**Group 3**	23.6 ± 7	80 ± 49.5	< 0.001^b^	12.5 ±3 .1	10.5 ± 2.3	< 0.001^b^	3.6 ± 0.9	2 ± 0.8	< 0.001^b^
**Group 4**	20.8 ± 10.6	82.6 ± 50.1	< 0.001^b^	13.5 ± 3.5	11.7 ± 3.1	< 0.001^b^	3.4 ± 1.4	2.2 ± 1.3	0.001^b^
**Group 5**	22.7 ± 11.7	83.8 ± 29.6	< 0.001^b^	13.2 ± 2.6	10.5 ± 2.5	< 0.001^b^	3.7 ± 0.9	2.3 ± 1	< 0.001^b^

Abbreviations: IELT, intravaginal ejaculation latency time; PEP, premature ejaculation profile; SD, standard deviation; QoL, quality of life

^b^Statistical significant P value

**Figure 1.**
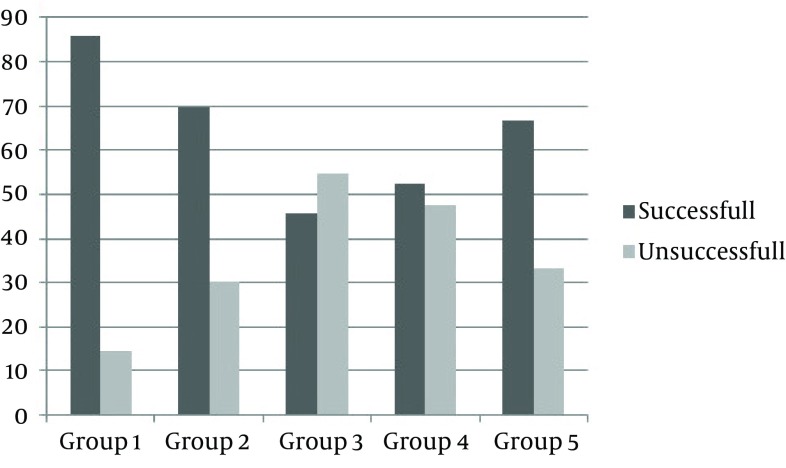
Success Rate of Treatment in Groups.

In this series, the success rate of treatment in PE with silodosin was 85.7% (Group 1), 69.6% for tamsulosin (Group 2), 45.5% for alfuzosin (Group 3), 52.4% for terazosin (Group 4), and finally 66.7% for doksazosin (Group 5) (Figure 1). Additionally there were statistically more increase in IELT and decrease in PEP scores in Group 1 compared to other groups, in statistical analyses (Table 3). However there were improvements in QoL for all groups, there was no statistically difference in pairwise comparison of groups (Table 3).

**Table 3.** Comparisons of Intravaginal Ejaculation Latency Time, Premature Ejaculation Profile, and Quality of Life in Groups[Table-fn fn5258]

Parameters	P Values for Pairwise Comparisons									
	Group 1				Group 2			Group 3		Group 4
	Group 2	Group 3	Group 4	Group 5	Group 3	Group 4	Group 5	Group 4	Group 5	Group 5
**Base IELT (second)**	0.28	0.14	0.6	0.31	0.86	0.53	0.93	0.31	0.77	0.57
**Post Treatment IELT (second)**	< 0.001^b^	< 0.001^b^	< 0.001^b^	< 0.001^b^	0.44	0.57	0.53	0.86	0.76	0.93
**Base PEP**	0.15	0.94	0.31	0.38	0.15	0.81	0.52	0.32	0.4	0.77
**Post Treatment PEP**	< 0.001^b^	< 0.001^b^	< 0.001^b^	< 0.001^b^	0.06	0.85	0.08	0.17	0.98	0.19
**Base QoL**	0.24	0.9	0.48	0.85	0.35	0.91	0.23	0.56	0.78	0.43
**Post Treatment QoL**	0.49	0.85	0.68	0.4	0.39	0.85	0.94	0.58	0.31	0.8

Abbreviations: IELT, intravaginal ejaculation latency time; NA, not assessed; PEP, premature ejaculation profile; QoL, quality of life

^b^Statistically significant P Value

Although there were four systemic adverse events in all patients, no patient did give-up treatment. Two patients had postural hypotension in Group 4 (treatment with terazosin). Postural hypotension was in the first week of treatment and it was disappeared in follow-up. Moreover a patient in Group 5 and a patient in Group 3 had dizziness. In follow-up dizziness was disappeared. Five patients in Group 1 and three patients in Group 2 had anejaculation. Anejaculation did not make any discomfort for these patients.

## 5. Discussion

In this pilot clinical trial, we evaluated the effectiveness of alpha blockers in the treatment of PE. Statistical significant development for PE’s parameters such as IELT, PEP and QoL scores were provided in all groups. Notably, silodosin seemed to be more successful in the treatment of PE than other alpha blocker agents. This may be related to super selective alpha receptor blockage with silodosin, especially in seminal vesicles ( 10 , 11 ). Additionally, silodosin is more selective for alpha-1-a receptors than other alpha blocker agents in prostate ( 10 , 11 ). In a recent study, Kobayashi et al. reported reduced seminal emission by silodosin ( 12 ). Furthermore alpha blocker agents inhibited the first step of ejaculation in all groups. In our opinion success rate in the treatment of PE was parallel to specificity of alpha blocker drugs in alpha 1-a receptor blocking. As we mentioned above, the best response to treatment was obtained in Group 1 (Table 3). Chronic usage of SSRIs and when needed topical anesthetics are acceptable treatment options in PE. However these treatment options have been used worldwide for PE, we investigated alpha blockers in PE ( 5 ). Recently, dapoxetine which is a SSRI has been accepted as the only drug approved for the on-demand treatment of PE in Europe ( 8 ). It has been just approved by Turkish Ministry of Health for the treatment of PE, but there has not been reported data about it in Turkey. Additionally, other SSRIs are being used for the treatment of PE in Turkey. Briefly, the mechanism of SSRIs is based on blocking serotonin reuptake and leads to higher synaptic serotonin levels in chronic usage than acute treatment ( 13 ). This is resulted in a clinical relevant delay of ejaculation ( 14 ). However SSRIs were not designed for PE they have been offered as continuous usage for PE except dapoxetine ( 5 ). Furthermore it is not clear that which usage mode of dapoxetine is more effective whether continuous or situational in the management of PE. Unlike the other SSRIs, dapoxetine is used 1-3 hours before sexual intercourse and chronic usage may not be necessary ( 14 ). Dapoxetine stands out without chronic usage for PE. However SSRIs have been approved for the treatment of PE in Europe there are some side effects ( 8 ). Although some of the side effects such as nausea, dry mouth, drowsiness, and reduced libido may be acceptable, serious complications such as mania may occur with SSRIs ( 15 ). Moreover seratonergic syndrome which can have a changing clinic from headache, nausea, sweating to delirium and coma may occur when concomitant use of monoamine oxidase inhibitors and lithium ( 5 ). Suicide attempt may be come into question as the worst side effect while SSRIs are being used chronically ( 16 ). Severe and serious side effects with alpha blockers have not been reported like SSRIs. In our series, the serious systemic side effects were postural hypotension and dizziness. Additionally these did not cause to give-up treatment and were not life-threatening. These clinical acceptable side effects with alpha blockers may provide us a safe option for the treatment of PE. 

Topical anesthetics work for reduction of penile sensation (17). However no systemic side effect has been reported with topical anesthetics, it is difficult to get used to perform and application. Additionally, residual topical anesthetics on penis may diffuse into vaginal wall. This may result in numbness in the partner (18). Continuous use of alpha blockers was easy to compliance in our series. Additionally they did not cause any side effect in partners of patients. PE treatment with alpha blockers is reported in previous studies (19, 20). Basar et al. reported terazosin in treatment of PE (20). Our results were parallel to them. In this series terazosin was used in Group 4. However Basar et al. (20) reported over 60% success in the treatment of PE with terazosin, we determined the success rate as 50% in Group 4. This may be related to shorter usage of terazosin. In their series, patients used terazosin at least for one month; however, in our series Group 4 used terazosin at least for two weeks. Cavallini reported that terazosin and alfuzosin might be used for patients with PE who did not respond to the psychological approach (19). In our series Group 3 used alfuzosin and 44.4% success was provided at the end of the short treatment period. However Cavallini (19) reported a 50% success with alfuzosin and terazosin, in our series the success rate with alfuzosin was less. This may be related with shorter treatment period again. Additionally in our series only three patients were refractory to psychotherapy. Despite all success rate 50% with terazosin and 44.4% with alfuzosin, statistical significant increase in IELT, decrease in PEP scores and progressions in QoL were provided. To our best knowledge there was no study about QoL with alpha blocker treatment in PE. Positive progressions in QoL may be provided as a result of increasing IELT and decreasing PEP. Notably more successful results were obtained with silodosin in Group 1, in this series. In a recent study, Sato el al. reported treatment of PE with silodosin (21). Additionally silodosin was used 2-3 hours before sexual intercourse in their study (21). This usage mode was similar to dapoxetine. In our study, we used daily continuously even up to 2 weeks for optimization of groups and drug usage. Although Sato et al. treated all patients with silodosin, there were some side effects such as anejaculation (21). These results were similar to our series. Five patients in Group 1 and 3 patients in Group 2 had anejaculation but there was no PE in these patients. Additionally there were no systemic adverse events in these patients. Although alfuzosin, terazosin and doksazosin may be used in the treatment of PE, systemic side effects should be kept in mind. Silodosin seemed to be safer than other alpha blocker agents for systemic adverse events in our series. In a recent investigation PE was categorized into four parts such as lifelong, acquired, natural variable PE, and premature-like ejaculatory dysfunction (22). There was no lifelong PE in our series. This was parallel to the report by Serefoglu et al. (22). Therefore in the light of our series, it seems that alpha blockers may be useful in PE treatment, except lifelong PE. There were some limitations in this series. Numbers of participants were low. Additionally we did not have long term follow-up data. We did not aim to prove the usage of alpha blockers in the treatment of all patients with PE in this study. In the light of our series, alpha blockers may be used in the treatment of PE in selected patients such as patients who do not have lifelong PE and who do not want to use SSRIs or local therapeutic agents. Additionally our study is unique in which more than two alpha blockers were compared for the treatment of PE. In all study groups, QoL development, increase in IELT and decrease in PEP scores were obtained. None of the patients did give up treatment with alpha blocker, and there was no serious side effect such as suicide attempt. In the light of literature and to our clinical aspect, silodosin may be an alternative of dapoxetine (21). More studies are needed in this respect. Therefore our pilot clinical trial study may be a pathfinder in this field. Silodosin may be used like dapoxetine as 2-3 hours before sexual intercourse. This was similar to the report by Sato et al. (21). However silodosin may not be effective as dapoxetine in PE, it does not cause severe systemic side effects (21).

Alpha blocker drugs seem to be able to prevent PE. However they provided development in QoL, increased IELT, and decreased PEP scores; silodosin was more successful than other alpha blocker agents. This may be a reflection of super selective blockage of alpha 1-a receptors. Additionally lower acceptable adverse events such as anejaculation and more efficiency are some of the prominent features of silodosin in PE. More comprehensive studies with long follow-up periods on this issue by holding the light of findings in this study may provide better treatment modalities for PE in near future.

## References

[1] Aschka C, Himmel W, Ittner E, Kochen MM (2001). Sexual problems of male patients in family practice.. J Fam Pract..

[2] Metz ME, Pryor JL, Nesvacil LJ, Abuzzahab F, Sr, Koznar J (1997). Premature ejaculation: a psychophysiological review.. J Sex Marital Ther..

[3] Rosen RC, Althof S (2008). Impact of premature ejaculation: the psychological, quality of life, and sexual relationship consequences.. J Sex Med..

[4] Arnold M, Ropert M (1981). Diagnostic and Statistical Manual of Mental Disorders..

[5] Montague DK, Jarow J, Broderick GA, Dmochowski RR, Heaton JP, Lue TF (2004). AUA guideline on the pharmacologic management of premature ejaculation.. J Urol..

[6] Master VA, Turek PJ (2001). EJACULATORY PHYSIOLOGY AND DYSFUNCTION.. Urologic Clinics of North America..

[7] Kara H, Aydin S, Agargun MY, Odabas O, Yilmaz Y (1996). The Efficacy of Fluoxetine in the Treatment of Premature Ejaculation: A Double-Blind Placebo Controlled Study.. J Urol..

[8] Hatzimouratidis K, Amar E, Eardley I, Giuliano F, Hatzichristou D, Montorsi F (2010). Guidelines on male sexual dysfunction: erectile dysfunction and premature ejaculation.. Eur Urol..

[9] de Almeida Kiguti LR, Pupo AS (2012). Investigation of the effects of alpha1-adrenoceptor antagonism and L-type calcium channel blockade on ejaculation and vas deferens and seminal vesicle contractility in vitro.. J Sex Med..

[10] Roehrborn CG (2009). Efficacy of alpha-Adrenergic Receptor Blockers in the Treatment of Male Lower Urinary Tract Symptoms.. Rev Urol..

[11] Kawabe K, Yoshida M, Homma Y (2006). Silodosin, a new alpha1A-adrenoceptor-selective antagonist for treating benign prostatic hyperplasia: results of a phase III randomized, placebo-controlled, double-blind study in Japanese men.. BJU Int..

[12] Kobayashi K, Masumori N, Hisasue S, Kato R, Hashimoto K, Itoh N (2008). Inhibition of Seminal emission is the main cause of anejaculation induced by a new highly selective alpha1A-blocker in normal volunteers.. J Sex Med..

[13] Giuliano F, Clement P (2006). Serotonin and premature ejaculation: from physiology to patient management.. Eur Urol..

[14] Pryor JL, Althof SE, Steidle C, Rosen RC, Hellstrom WJG Efficacy and tolerability of dapoxetine in treatment of premature ejaculation: an integrated analysis of two double-blind, randomised controlled trials.. The Lancet..

[15] Balachandra K (2001). RE: TREATMENT OF PREMATURE EJACULATION WITH PAROXETINE HYDROCHLORIDE AS NEEDED: 2 SINGLE-BLIND PLACEBO CONTROLLED CROSSOVER STUDIES.. J Urol..

[16] Tamam L, Ozpoyraz N (2002). Selective serotonin reuptake inhibitor discontinuation syndrome: A review.. Advances in Therapy..

[17] Atikeler MK, Gecit I, Senol FA (2002). Optimum usage of prilocaine-lidocaine cream in premature ejaculation.. Andrologia..

[18] Sahin H, Bircan MK (1996). Re: Efficacy of Prilocaine-Lidocaine Cream in the Treatment of Premature Ejaculation.. J Urol..

[19] Cavallini G (1995). Alpha-1 blockade pharmacotherapy in primitive psychogenic premature ejaculation resistant to psychotherapy.. Eur Urol..

[20] Basar MM, Yilmaz E, Ferhat M, Basar H, Batislam E (2005). Terazosin in the treatment of premature ejaculation: a short-term follow-up.. Int Urol Nephrol..

[21] Sato Y, Tanda H, Nakajima H, Nitta T, Akagashi K, Hanzawa T (2012). Silodosin and its potential for treating premature ejaculation: a preliminary report.. Int J Urol..

[22] Serefoglu EC, Yaman O, Cayan S, Asci R, Orhan I, Usta MF (2011). Prevalence of the complaint of ejaculating prematurely and the four premature ejaculation syndromes: results from the Turkish Society of Andrology Sexual Health Survey.. J Sex Med..

